# Case Report: Transoral Endoscopic Thyroidectomy *via* Vestibular Approach in Pediatric Thyroid Cancer

**DOI:** 10.3389/fped.2021.765278

**Published:** 2021-10-26

**Authors:** Duy Q. Ngo, Duong T. Le, Giap N. Hoang, Quy X. Ngo, Quang V. Le

**Affiliations:** ^1^Department of Head and Neck Surgery, Vietnam National Cancer Hospital, Hanoi, Vietnam; ^2^Department of Oncology, Hanoi Medical University, Hanoi, Vietnam

**Keywords:** TOETVA, pediatric thyroid cancer, thyroid cancer in children, transoral approach, transoral thyroidectomy

## Abstract

**Background:** Transoral endoscopic thyroidectomy via vestibular approach (TOETVA) is a new technique that has become more popular worldwide because of its many advantages. However, this novel approach for thyroid cancer treatment in children is highly challenging, even for high-volume surgeons. In our study, we report our experiences with TOETVA for pediatric patients with thyroid cancer.

**Patients and Methods:** This study included four pediatric patients who underwent TOETVA performed by a single surgeon between June and December 2020. Patient demographics and surgical outcomes including operative time, incidence of complications, and length of hospital stay were evaluated.

**Results:** Four patients successfully underwent TOETVA with no complications. All patients were girls, aged from 13 to 18. Three patients underwent lobectomy and isthmusectomy, plus prophylactic unilateral central neck dissection. One patient had a total thyroidectomy, plus prophylactic bilateral central neck dissection. The mean operative time was 85 min for the lobectomy and 120 min for total thyroidectomy plus central neck dissection. The median hospital stay was 4.1 days. No drains were used. The histological examination showed four cases of malignant disease (papillary thyroid carcinoma). The mean number of harvested lymph nodes was 4.2 (ranged 3 to 8).

**Conclusion:** In the hands of a high-volume surgeon, TOETVA is a novel, feasible, and safe approach for treating selected pediatric patients with thyroid cancer.

## Introduction

Thyroid nodules are less common in children, but this age group has witnessed an increased rate of thyroid cancer (1). Surgery plays a major role in treatment. The prognosis for children with thyroid cancer is excellent, with a high survival rate after treatment (1, 2). Nevertheless, a cervical scar has been shown to have an impact on the confidence and the quality of life of children experiencing thyroid surgery, especially female patients (3). Thus, several new approaches to surgical methods have been introduced to reduce the risk of cervical scar (4, 5).

Trans-oral endoscopic thyroidectomy via vestibular approach (TOETVA) is a new technique, with the aesthetic result of truly scar-free healing and minimally invasive dissection. It also offers an accessible approach to both thyroid lobes and facilitates the removal of neck lymph nodes (6–8). Thus, TOETVA is becoming more popular. However, while there is extensive research on TOETVA for adults, there is little on the pediatric population. In our study, we present the first case series of TOETVA used with children in Vietnam.

## Patients and Methods

Following the ATA guidelines, all patients included in this study were ≤ 18 yr old (1). Between June and December 2020, four patients were admitted to the Department of Head and Neck Surgery and selected for the transoral approach. All these patients had a preoperative assessment, including thyroid hormonal level tests, neck ultrasound examination, and fine-needle aspiration (in line with The Bethesda System for Reporting Thyroid Cytopathology—TBSRTC) (9).

Inclusion criteria for TOETVA were as follows: (1) thyroid nodule (TBSRTC II–IV) ≤ 6 cm in size; (2) thyroid nodule (TBSRTC V–VI) ≤ 2 cm in size with no lymph node involvement; (3) total size of thyroid gland ≤ 10 cm in diameter per lobe (10, 11).

Contraindications for TOETVA included (1) previous anterior neck surgery, (2) tracheal or esophageal invasion, (3) previous radiation to the head or neck, (4) recurrent laryngeal nerve palsy, (5) oral cavity infection, (6) uncontrolled hyperthyroidism (10, 11), and (7) patients under 10 years of age or less than 30 kg of weight.

## Surgical Technique

**Figure 1:**
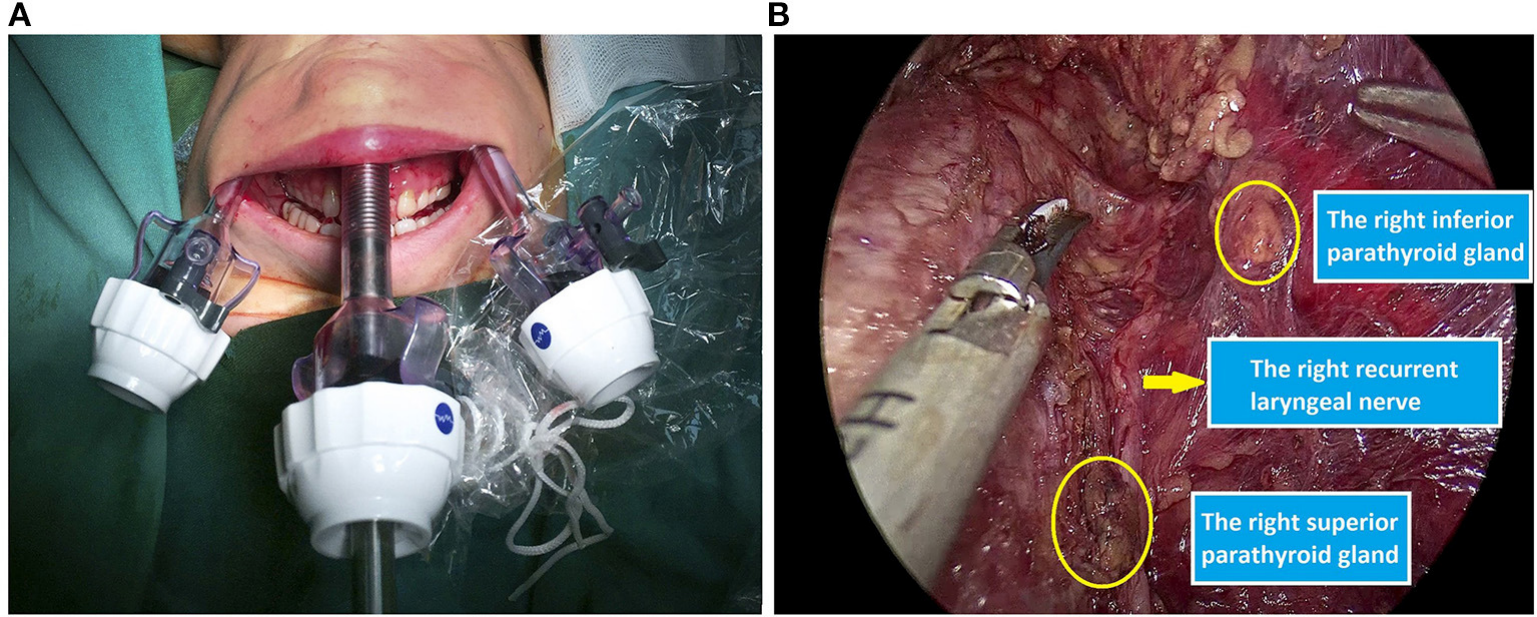
Intraoperative images. **(A)** Trocar set-up. **(B)** Preservation of the recurrent laryngeal nerve and parathyroid glands.

The surgical steps have been described in previous publications and are based on Anuwoong's technique (6). In brief, the patients were placed in a supine position. After intubation, three incisions were made in the oral vestibule for the insertion of endoscopic 5–10 mm trocars. To insert a 30-degree endoscope, a 5-mm central trocar is recommended, especially for pediatric patients. Working space was then created by using a hook cautery and/or an ultrasonic scalpel. Next, the strap muscles were retracted laterally with a transcutaneous suture. The pyramidal lobe was dissected and separated from the trachea. The isthmus was then divided, and the superior thyroid vessels were dissected and divided using an energy device. The upper parathyroid gland and then the recurrent laryngeal nerve (RLN) were identified. The thyroid lobe was dissected from the trachea and the RLN while preserving the lower parathyroid. The specimen was put into an Endo Catch bag and removed via a central incision (Figure 1).

## Results

A summary of the preoperative data for the four patients is presented in Table 1. All the patients were girls, aged from 13 to18. Three patients underwent lobectomy and isthmusectomy plus prophylactic unilateral central neck dissection. One patient had a total thyroidectomy plus prophylactic bilateral central neck dissection. The median hospital stay was 4.2 days. All the TOETVA procedures were successfully carried out, with no need for conversion to the open approach. In each pediatric TOETVA, the RLNs and parathyroid glands were visualized while performing the dissection.

**Table 1:** Individual case characteristics and surgical outcomes.

**Patient**	**Gender**	**Age (years)**	**Tumor location**	**Tumor size (mm)**	**FNA (Bethesda)**	**Surgery**	**Operative surgery (mins)**	**Number of haversted LN**	**Number of positive LN**	**pT**	**pN**	**Complication**	**Follow up (months)**	**Recurrence**
Pt 1	Female	18	Left lobe	4 ×5	5	Hemithyroidectomy + uniteral CND	80	3	0	T1a	N0	None	14	No
Pt 2	Female	13	Left lobe	6 ×7	5	Hemithyroidectomy + uniteral CND	90	8	0	T1a	N0	None	15	No
Pt 3	Female	16	Right lobe	4 ×6	5	Hemithyroidectomy + uniteral CND	85	6	0	T1a	N0	None	15	No
Pt 4	Female	15	Right lobe	12 ×8	5	Bilateral thyroidectomy + bilateral CND	120	6	1	T1b	N1a	None	16	No

*Pt, Patient; CND, central neck dissection; FNA, Fine needle aspiration; LN, Lymph nodes*.

**Figure 2:**
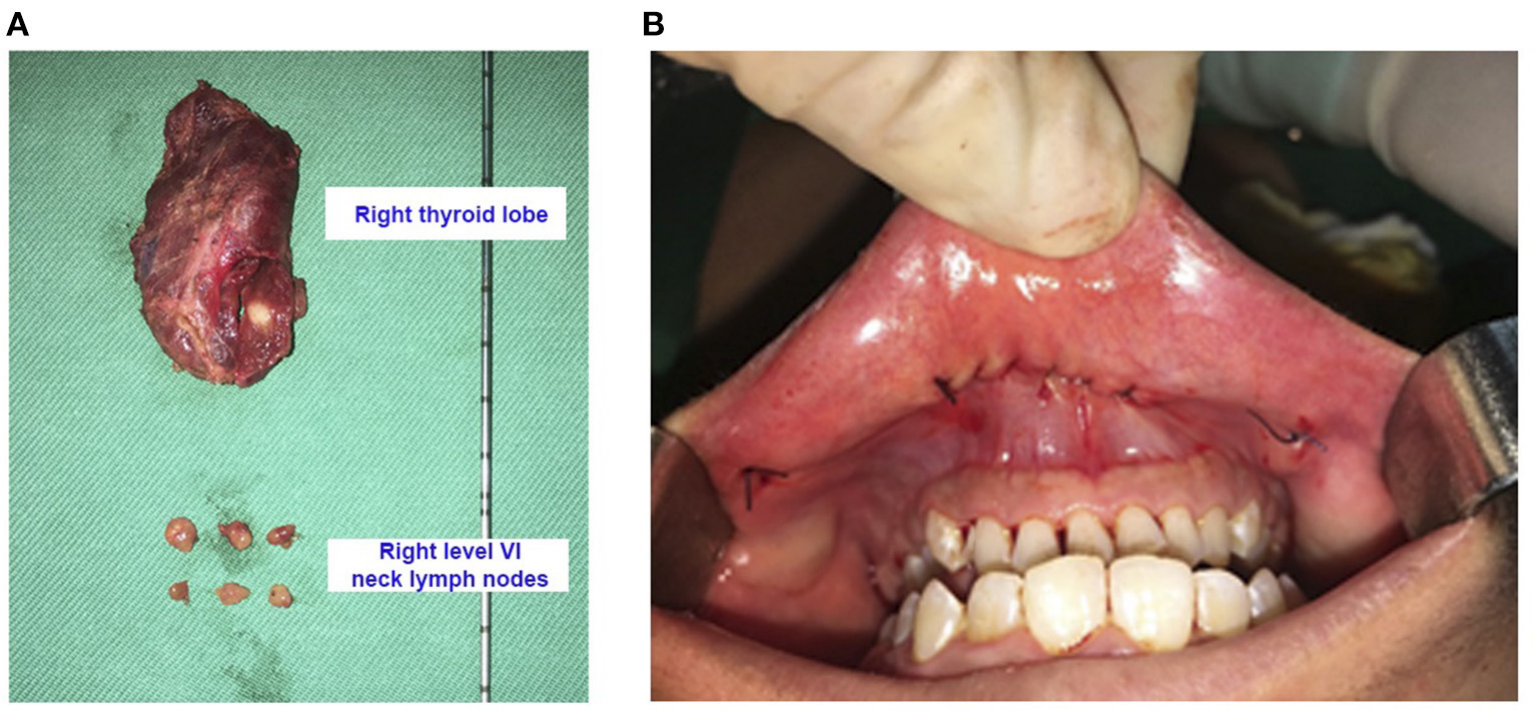
**(A)** Surgical specimens after right hemithyroidectomy and right central neck dissection (16-year-old female patient with thyroid cancer of the right lobe pT1aN0M0). **(B)** The incisions were closed by two layers with absorbable interrupted sutures.

The mean operative time was 85 min (range 80–90 min) for lobectomy and isthmusectomy plus central neck dissection, while the time for total thyroidectomy plus central neck dissection was 120 min. No drains were used (Figure 2).

**Figure 3:**
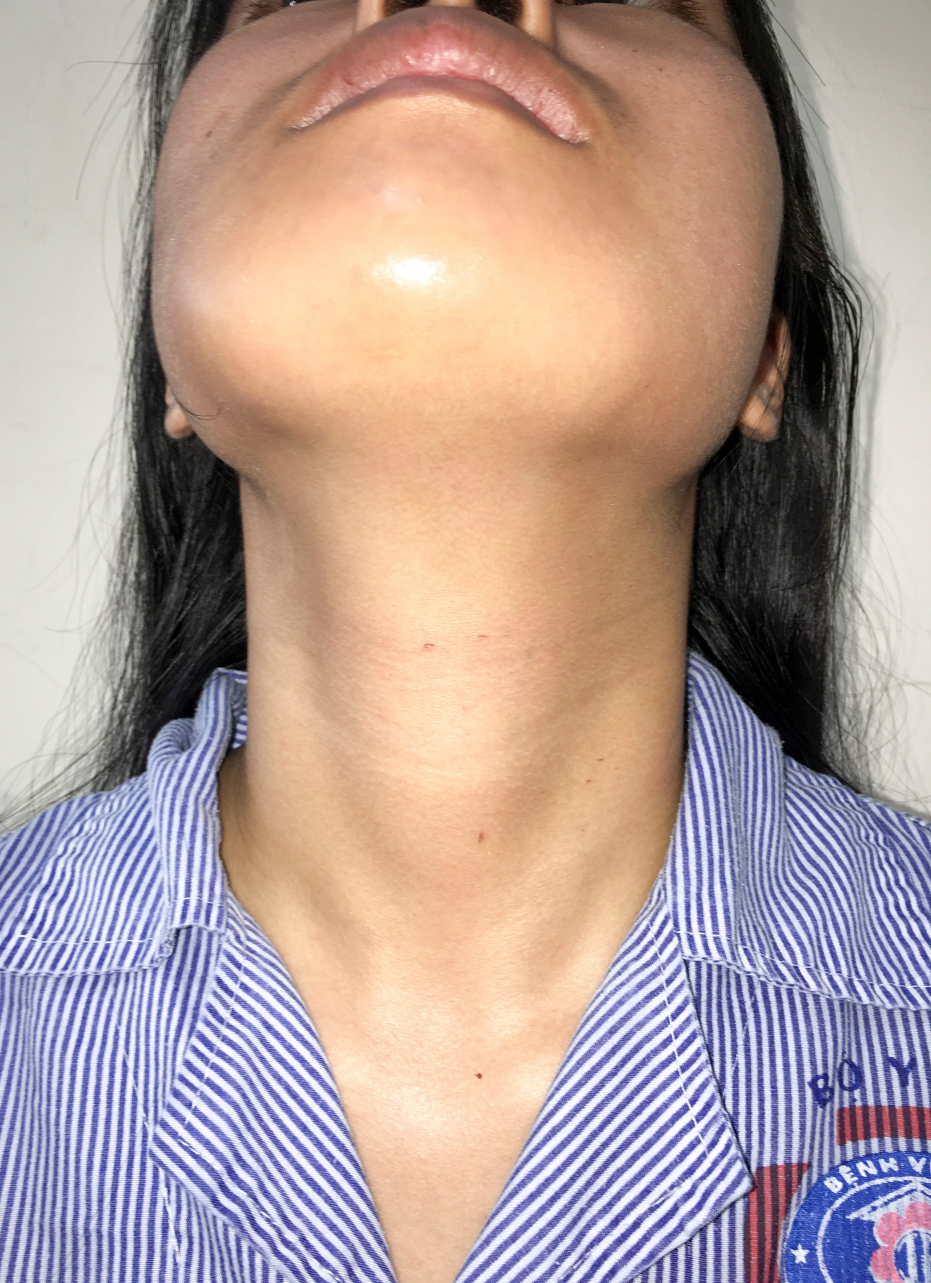
Cosmetic results after surgery 5 days (16-year-old female patient with thyroid cancer of the right lobe pT1aN0M0).

All patients were performed successfully via transoral approach without any surgical complication. No permanent or transient complications were documented, including RLN injury and hypocalcemia. No transient mental nerve injury or chin hypoesthesia was observed. There was no occurrence of skin injury, bleeding, seroma, or infection or need for reoperation in any of the patients. All pediatric patients were completely satisfied with the cosmetic results (Figure 3).

The histological examination showed four cases of malignant disease (papillary thyroid carcinoma). The mean number of harvested lymph nodes was 4.2 (range 3–8), while there was only one patient with positive lymph nodes (1/6 positive lymph nodes). All tumors were completely excised with negative margins.

After a median follow-up of 14.25 (range 10–16) months, none of these patients presented evidence of recurrent disease and any surgical complication related to transoral endoscopic thyroidectomy.

## Discussion

Pediatric thyroid cancer is rare in children, accounting for approximately 1.5% of all cancers in the under-18 age group, with 4.8–5.9 cases per million children annually and an increasing trend (1, 2). The major pathology of pediatric patients is a well-differentiated thyroid carcinoma. However, the prognosis of the group is excellent, with high survival rates (12). Thus, quality of life should be considered when choosing a surgical approach. With traditional open surgery, a neck scar following thyroidectomy leads to a decline in quality of life in children (3). A cervical scar is associated with an increased risk of depression in the pediatric population. Hence, different approaches have been developed to avoid the neck incision in children (4, 5). TOETVA is a new technique with many advantages, including an aesthetic result with scar-free healing. TOETVA has become popular worldwide (7, 10, 13). However, there has been only limited reported use in the pediatric population; thus, investigation of the feasibility and safety of TOETVA for use with this population is needed. To the best of our knowledge, this is the second case series for TOETVA for this group.

According to ATA guidelines, pediatric patients with thyroid cancer should be treated with a total thyroidectomy because of the risk of contralateral lobe cancer (1). However, a recent study by Tate Nice of the 15-yr follow-up of 4,000 pediatric patients showed that total thyroidectomy did not improve overall survival compared with a lobectomy in the low-risk group, which is similar to adults (14). However, despite the higher cervical lymph node metastatic rate in children compared with adults, prophylactic central neck dissection was found to increase the fatality risk in children due to the complications of hypoparathyroidism and RLN injury (1, 15, 16). Thus, indications for prophylactic central neck dissection should be considered carefully to balance the merits and risks. Thus, in cT1,2N0M0 patients with a unifocal disease, we performed the ipsilateral prophylactic central neck dissection and made a frozen section of level VI lymph nodes. If the result was positive, contralateral central neck dissection was carried out (15). In our case series, three patients with unifocal disease underwent lobectomy and unilateral prophylactic central neck dissection, and one patient underwent total thyroidectomy plus bilateral prophylactic central neck dissection because the results of the intraoperative frozen section of level VI lymph nodes were positive.

Thyroid surgery is uncommon in the pediatric population. Thus, there may be an increased percentage with surgical complications. A study of 464 pediatric patients who had undergone a thyroidectomy showed that the most common postoperative complication was temporary hypoparathyroidism, at 37% of patients, following by temporary RLN injury at 2.37% (17). The percentage of permanent hypoparathyroidism or RLN injury is less than 1%. Oden Cohen reported on 48 pediatric patients who had undergone thyroidectomy via a transoral approach; 33% experienced temporary hypoparathyroidism and 1.6% temporary RLN injury (18). No patient suffered permanent RLN injury or hypoparathyroidism.

Our study showed similar results, as no patient suffered temporary hypoparathyroidism or any other complications, such as RLN injury, mental nerve injury, hemorrhage, or seroma. Overall, we achieved good results in all four cases, with no long-term complications including recurrent laryngeal nerve injury or hypoparathyroidism. Although our report demonstrated good short-term outcomes, the pediatric patients needed to be followed to confirm longer-term oncological results. On the other hand, thyroid surgery in children is more difficult than in adults. Thus, we highly recommended that TOETVA in the pediatric population should only be performed by high-volume thyroid surgeons.

## Conclusion

TOETVA is a new, feasible, and safe approach for pediatric patients with thyroid cancer. We recommend that only high-volume thyroid surgeons with experience of TOETVA could perform TOETVA on pediatric patients.

## Data Availability Statement

The original contributions presented in the study are included in the article/supplementary material, further inquiries can be directed to the corresponding author/s.

## Ethics Statement

The studies involving human participants were reviewed and approved by Local Institutional Review Board. Written informed consent to participate in this study was provided by the participants' legal guardian/next of kin.

## Author Contributions

DN and QL study conception and design. QN and DL data acquisition. DN, QN, and GH analysis and data interpretation. DN and GH drafting of the manuscript. QL critical revision. DL submit this form with the manuscript. All authors have read and agreed to the published version of the manuscript.

## Funding

DN was funded by Vingroup Joint Stock Company and supported by the Domestic Master/PhD Scholarship Programme of Vingroup Innovation Foundation (VINIF), Vingroup Big Data Institute (VINBIGDATA), code [VINIF.2020.TS.11].

## Conflict of Interest

The authors declare that the research was conducted in the absence of any commercial or financial relationships that could be construed as a potential conflict of interest.

## Publisher's Note

All claims expressed in this article are solely those of the authors and do not necessarily represent those of their affiliated organizations, or those of the publisher, the editors and the reviewers. Any product that may be evaluated in this article, or claim that may be made by its manufacturer, is not guaranteed or endorsed by the publisher.
